# Carbohydrate restriction drives greater perturbations in circulating metabolites than low energy availability in elite male athletes

**DOI:** 10.14814/phy2.70752

**Published:** 2026-02-03

**Authors:** Kyle A. Dunlop, Nathan G. Lawler, Jamie Whitfield, Alannah K. A. McKay, Nicolin Tee, Megan L. Ross, Stacey N. Reinke, John A. Hawley, David Broadhurst, Louise M. Burke

**Affiliations:** ^1^ Human Performance & Metabolism Centre, Mary MacKillop Institute for Health Research Australian Catholic University Melbourne Victoria Australia; ^2^ School of Science Edith Cowan University Perth Western Australia Australia; ^3^ Department of Sport and Exercise Sciences, Institute of Sport Manchester Metropolitan University Manchester UK; ^4^ Present address: Centre for Computational and Systems Medicine, Health Futures Institute, Harry Perkins Institute Murdoch University Perth Western Australia Australia

**Keywords:** carbohydrate metabolism, lipid metabolism, low energy availability, mass spectrometry, metabolomics

## Abstract

Periods of low energy availability (LEA) are common in elite athletes and typically arise from reduced energy intake, often involving some degree of carbohydrate (CHO) restriction. Whether the metabolic profile created by energy restriction per se is distinct compared to that driven by CHO restriction is unknown. Using untargeted metabolomics, we examined metabolic perturbations linked to CHO restriction and energy restriction in plasma from elite male endurance athletes. In a semi‐randomized controlled trial, athletes (*n* = 20) completed one of three 5‐day dietary interventions: high energy‐high CHO (HCHO); LEA (energy‐restricted, CHO‐reduced); or low‐CHO, high‐fat (LCHF; energy‐matched, CHO‐restricted). Plasma samples were taken at multiple timepoints pre‐ and post a standardized 25 km race walk protocol. Metabolomic analysis was performed using liquid chromatography–mass spectrometry (LC–MS), with multivariate analysis conducted using RM‐ASCA+ and hierarchical clustering. A total of 5391 metabolic features were detected and 138 metabolites annotated. LCHF induced substantial metabolic perturbations, especially after prolonged exercise, including elevations in fatty acyls, hydroxy acids, dicarboxylic acids and acylcarnitine intermediates, responses not seen under LEA. We conclude that CHO restriction concomitant with a high‐fat load induces a greater metabolic perturbation in selected lipid‐based metabolites than short‐term LEA exposure in elite athletes undergoing prolonged endurance exercise.

## Graphical abstract

Elite male race walkers underwent a 5 d dietary intervention of either high carbohydrate (HCHO), low‐carbohydrate high‐fat (LCHF), or low energy availability (LEA) to evaluate their metabolomic response. Following a standardized 25 km endurance walk, blood samples were collected across five timepoints, ranging from a fasted state to 3 h post‐exercise. Following plasma processing and analysis via mass spectrometry, data interpretation revealed that carbohydrate restriction (LCHF) may serve as a potent driver of circulating metabolite shifts than LEA. This is evidenced by marked perturbations in pathways related to fatty acid β‐oxidation and increased acylcarnitine production, which distinguish the LCHF profile from both the LEA and HCHO conditions.

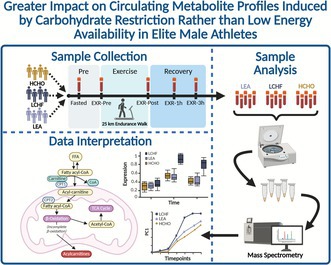

## 1 INTRODUCTION

As part of varied training and/or nutritional strategies undertaken to achieve optimal performance, many elite athletes involved in weight‐sensitive and/or endurance‐based sports periodically undertake deliberate periods of reduced energy availability (EA). While energy restriction can happen unintentionally, here it occurs because of planned reductions in energy intake (EI) and/or increased exercise energy expenditures (EEE) in order to manipulate body composition for specific competitions (Martin‐Rodriguez et al., 2024). Such practices may result in periods of low energy availability (LEA), which is often characterized by a downregulation of multiple biological systems in several tissues and organs due to insufficient energy to meet daily bodily requirements (Loucks et al., 2011). Although this practice is embedded into athletic preparation and is often deemed a crucial element in achieving sporting success, prolonged exposure may lead to problematic scenarios in which the metabolic perturbations are no longer transient or reversible (Mountjoy et al., 2023). Indeed, prolonged LEA exposure is associated with a variety of health‐related disorders, including reproductive dysfunction, impaired bone health, gastrointestinal dysfunction, and perturbations in glucose/lipid handling (Mountjoy et al., 2023). The constellation of these symptoms has been termed Relative Energy Deficiency in Sport (REDs) (Mountjoy et al., 2023), with recent attention focused on gaining a better understanding of the mechanisms that trigger the dysfunction of bodily systems, and the characteristics of LEA exposure that become problematic (Burke, Ackerman, et al., 2023).

Although the relative reduction in available energy is often promoted as the key hallmark of LEA and the driver of subsequent metabolic changes, experimental trials investigating LEA also include a relative and/or absolute reduction in carbohydrate (CHO) intake and availability to total EI (Jurov et al., 2022; Martin et al., 2021; McKay, Peeling, et al., 2022). Furthermore, recent observations suggest that CHO restriction may negatively impact body systems to a greater extent than insufficient energy alone (Fensham et al., 2022; McKay, Peeling, et al., 2022). Indeed, reducing CHO availability provokes marked effects on resting and exercise‐associated patterns of substrate utilization while also perturbing acute processes underlying gene expression and cell signaling (Hawley & Burke, 2010).

While many studies have investigated the effects of LEA on individual physiological systems (Loucks & Thuma, 2003; Martin et al., 2021; Oxfeldt et al., 2023; Papageorgiou et al., 2018), evidence from evolutionary biology, such as “The Life History Theory”, suggests that energy partitioning, and thus the presentation of LEA, differs markedly across individuals according to their characteristics and circumstances (Shirley et al., 2022). Accordingly, employing systems‐level analytical approaches may be required to capture the multidimensional biological responses to these dietary interventions. In this regard, mass spectrometry (MS)‐based metabolomics has emerged as a powerful platform to investigate perturbations in substrate availability and turnover caused by exercise or alterations in dietary intake (Brennan et al., 2018; Cipryan et al., 2023; Hawley & Hoffman, 2025; Lewis et al., 2010; Morville et al., 2020). Metabolomics is a relatively noninvasive technique, using samples collected from a variety of biofluids, and provides a “snapshot” of an individual's metabolic status (Belhaj et al., 2021). This makes it well suited for use in the athletics arena where EI and EEE can often fluctuate substantially across an athlete's training cycle, enabling identification of key regulatory signals that lie downstream of genomic, transcriptomic, proteomic, and environmental inputs. The purpose of this study was to utilize untargeted metabolomics to characterize the profile of plasma metabolites induced by severe CHO or energy restriction in elite male race walkers during a block of intense training.

## 2 MATERIALS AND METHODS

### 2.1 Ethical approval

This study is a secondary exploratory analysis from a previously published clinical trial (Burke et al., 2021), which was registered with the Australian New Zealand Clinical Trials Registry (ACTRN12618001974291p) and received ethical approval from the Human Research Ethics Committee of the Australian Institute of Sport (#20181203). All procedures adhered to the principles of the Declaration of Helsinki. Prior to participation, athletes were provided with a comprehensive explanation of the study, both verbally and in writing, before giving their written informed consent.

### 2.2 Participants and study design

**FIGURE 1:**
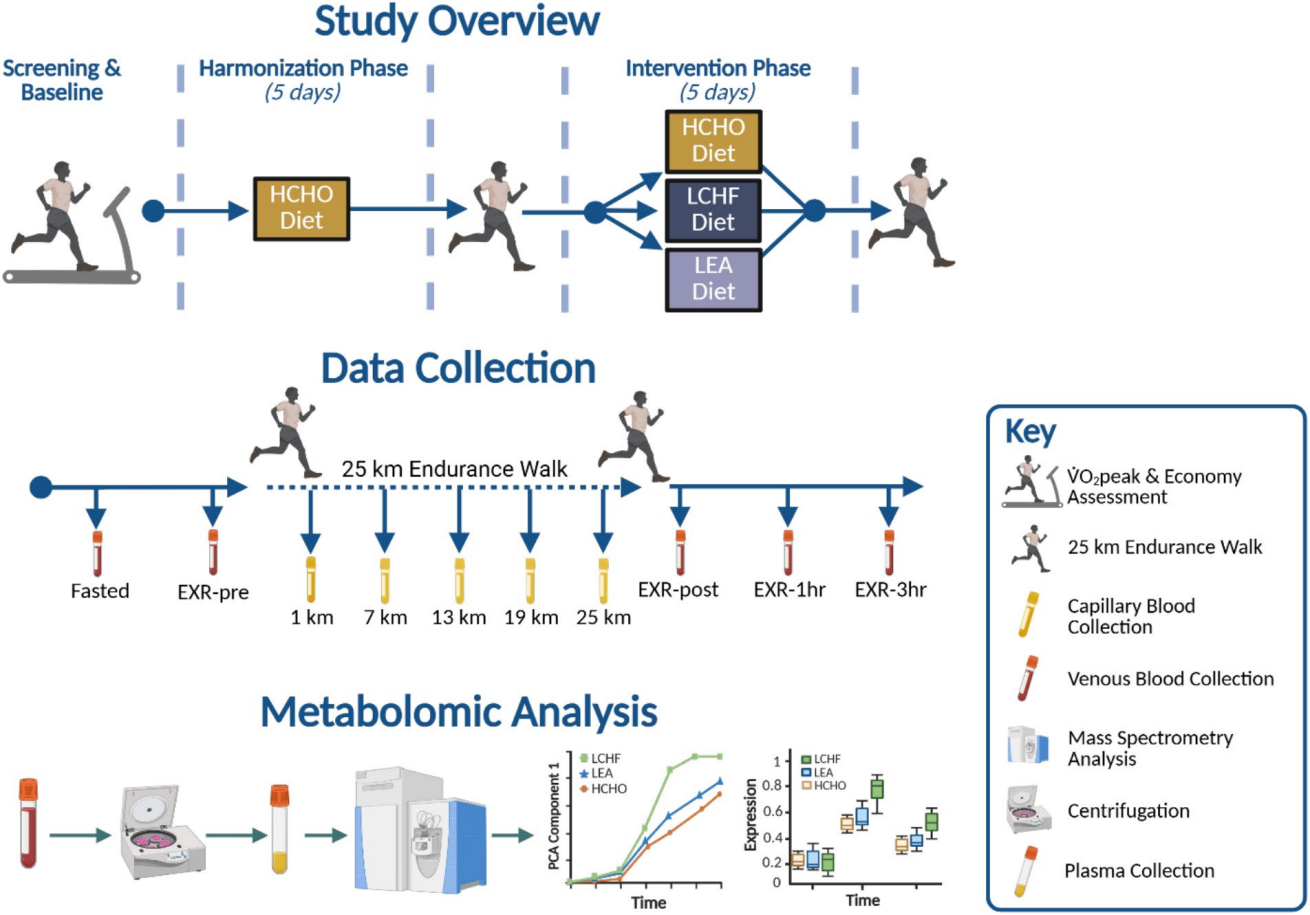
Schematic overview of the study design, data collection and metabolomic workflow. EXR‐1 h, 1 h into recovery; EXR‐3h, 3 h into recovery; EXR‐post, post‐exercise; EXR‐pre, pre‐exercise; HCHO, high energy, high carbohydrate; LCHF, low carbohydrate, high fat; LEA, low‐energy availability. Figure created in BioRender.

Twenty high performance male race walkers, ranging from Tier 3 (national level) to Tier 5 (world class) (McKay, Stellingwerff, et al., 2022), participated in this parallel group design (Figure 1). Following baseline testing to determine maximal and submaximal physiological capacity (Burke et al., 2021), athletes entered a “harmonization” period in which they consumed a high energy, high CHO (HCHO) diet involving high energy‐ and CHO‐availability for 5 days. This was undertaken to ensure athletes began the intervention period with adequate EA, minimizing the effects of prior dietary habits and allowing athletes at risk of REDs to be identified and excluded. Athletes then completed a hybrid lab/field 25 km endurance walk (detailed below) before commencing the ‘intervention’ phase in which they were allocated into either (1) HCHO diet (*n* = 6); (2) LEA diet (*n* = 7); or (3) low‐CHO, high fat (LCHF) diet (*n* = 7) for 5 days. The protocol for achieving this semi‐randomized allocation has been previously described (Burke et al., 2017) and justified, enabling a balance between maintaining athlete compliance/motivation and matching groups for age, body mass, maximal aerobic capacity, and 20 km race walk personal best times. Seven athletes participated in previous studies carried out by the research team (Burke et al., 2017, 2020), with one individual electing to repeat the LCHF intervention. Following the completion of the 5‐day intervention phase, athletes repeated the 25 km endurance walk protocol. Venous blood samples (2 mL) were collected prior to and following exercise, while fingertip capillary samples were collected during the exercise bout.

### 2.3 Dietary intervention

EA was defined according to the following formula (Loucks, 2004):

$$\mathrm{EA}=\frac{\left(\mathrm{EI}-\mathrm{EEE}\right)}{\mathrm{FFM}}$$

A DXA assessment of fat‐free mass (FFM) was undertaken using the Best Practice Protocols of the Australian Institute of Sport, involving standardized athlete presentation and positioning (Nana et al., 2015) on an iDXA (GE Healthcare, Milwaukee, WI, USA) and standardized image analysis (enCorev16, GE Healthcare). Daily EEE was derived from the planned training program for each athlete, with removal of measured values of resting metabolic rate from the energy expended during each session (McKay, Peeling, et al., 2022). Planned EI for each day was individually calculated for each athlete using the EA formula, with macronutrient contribution to EI being allocated according to a consistent formula for each diet (Table 1).

**TABLE 1:** Energy availability and macronutrient composition targets for each dietary condition.

Dietary characteristic	HCHO	LEA	LCHF
Daily intake			
EA (kcal·kg FFM^−1^·day^−1^)	40	15	40
Carbohydrates (%)	65	60	5–10
Carbohydrate (g·kg^−1^·d^−1^)	~9	~5	~0.5
Protein (%)	15	25	15
Protein (g·kg^−1^·d^−1^)	~2.2	~2.2	~2.2
Fat (%)	20	15	75–80
Fat (g·kg^−1^·d^−1^)	~1.3	~0.6	~5
Pre‐exercise meal			
Carbohydrate (g·kg^−1^)	2	1	< 0.1
Fat (g·kg^−1^)	<0.2	<0.1	~0.7
Intra‐exercise intake			
Carbohydrate (g·h^−1^)	60	30	< 2
Fat (g·h^−1^)	0	0	20
Fluid (mL·h^−1^)	600	600	600

Abbreviations: EA, energy availability; HCHO, high energy‐high carbohydrate; LCHF, low carbohydrate‐high fat; LEA, low‐energy availability.

All meals and fluids throughout the study were supplied by the research team and consumed in a free‐living environment in which dietary compliance was monitored. Menu and meal preparation were developed by a professional chef, sports dietitians and a food service dietitian. Meals were individually tailored for each athlete (Mirtschin et al., 2018). To help incorporate personal food preferences and nutritional needs within the daily EA limits, training‐related EEE and macronutrient goals. Food choices were altered in the latter half of the day in the event that daily EEE targets deviated from the prescribed exercise goal as described previously (Burke, Whitfield, et al., 2023).

### 2.4 Standardized 25 km endurance walk

Athletes performed a 25 km endurance walk after consuming a standardized meal ~2 h prior to commencing exercise in accordance with their respective dietary conditions and excluded caffeine (Burke et al., 2021). Key nutritional characteristics of these meals are summarized in Table 1, noting that HCHO and LCHF meals were isoenergetic. The test was conducted in a hybrid laboratory‐field manner, with 0–1, 6–7, 12–13, 18–19, and 24–25 km performed on a treadmill and the remaining distances performed on a closed outdoor loop course (~5 km). Fluid stations were provided every ~2 km, as in competition, with foods/sports food provided during each laboratory visit to supervise complete intake. Table 1 summarizes key nutritional characteristics of EI; athletes in the HCHO and LEA group consumed sports gels (optional and containing 50 mg caffeine) and water, while athletes in the LCHF group consumed electrolyte‐supplemented water and LCHF cookies. The treadmill speed was set at a speed corresponding to the pace corresponding to their best 50 km competition pace (i.e., either 12 or 13 km·h^−1^). The pace for the outside portion of the test was negotiated on an individualized basis, but athletes were required to repeat the same pace for both 25 km walking tests, which was monitored through the use of global positioning system (GPS) watches.

### 2.5 Blood sample collection

Fasted, pre‐exercise (2 h post‐meal; EXR‐pre), immediately post‐exercise (EXR‐post), 1‐ and 3‐h recovery (EXR‐1h and EXR‐3h, respectively) venous blood samples were collected via an indwelling forearm cannula. Plasma samples were collected from 2 mL vacuette lithium heparin tubes and used exclusively for metabolomic analysis. Serum samples for free fatty acids (FFA) analysis were collected from 4 mL vacuette SST tubes. Serum samples were allowed to clot for 30 min prior to centrifugation, which was performed alongside plasma samples at 1500*g* for 10 min at 4°C. Following centrifugation, plasma and serum samples were aliquoted and stored at −80°C for subsequent analysis. Capillary blood samples were collected throughout the 25 km endurance walk upon completing the treadmill portions of the test (1, 7, 13, 19, and 25 km) for analysis of lactate (Lactate Pro 2, Akray, Japan), glucose (FreeStyle Optium Neo, Abbott Diabetes Care, Doncaster, Victoria, Australia) and β‐hydroxybutyrate (βHB; FreeStyle Optium Neo) concentrations.

### 2.6 Sample preparation

Fasted, EXR‐pre, EXR‐post, EXR‐1h, and EXR‐3h sampling timepoints were selected for metabolomics analysis due to the acute effects of prolonged exercise on substrate availability and molecular signaling events (Burke et al., 2017, 2021; Odland et al., 1998; Watt et al., 2004). On the day of analysis, plasma samples (~300 μL) were thawed at 4°C and 50 μL was transferred and mixed with either 50 μL of ice‐cold liquid chromatography‐mass spectrometry (LC‐MS) grade methanol containing two internal standards, D5‐TRP (Cat. No. 615862, Cambridge Isotopes Laboratories) and taurocholate (Cat. No. T4009‐250 mg, Sigma‐Aldrich, St. Louis, MO) for reversed phase chromatography, or 150 μL of ice‐cold LC‐MS grade acetonitrile containing two internal standards: C13‐leucine (Cat. No. 490059‐1G, Sigma Aldrich, St. Louis, MO) and D8‐valine (Cat. No. 486027‐1G, Sigma Aldrich, St. Louis, MO) for hydrophilic interaction liquid chromatography (HILIC) as previously reported (Manaf et al., 2018). Samples were vortexed before centrifugation for 20 min at 1800 *g* at 4°C. The supernatant was then transferred to separate vials and placed in the autosampler at 6°C. A pooled quality control (PQC) was generated by combining equal volumes of each study sample that was analyzed throughout the run to evaluate analytical stability.

### 2.7 Liquid chromatography mass spectrometry

Analysis was performed using the Dionex UltiMate 3000™ platform, which includes an ultra‐high‐performance liquid chromatography pump coupled with a heated electrospray Q Exactive Focus Orbitrap mass spectrometer (Thermo Scientific, San Jose, CA, USA). Reversed‐phase separation was performed on a Hypersil GOLD column (100 × 2.1 mm, 1.9 μm particle size; Thermo Fisher Scientific, Runcorn, UK) with an in‐line filter. Sample analysis in both positive and negative ionization modes was performed using 0.1% formic acid in LC‐MS water (solvent A) and 0.1% formic acid in LC‐MS methanol (solvent B). The elution gradient was as follows: isocratic at 100% solvent A for 1 min, followed by an increase to 100% solvent B in 15 min and maintained at 100% solvent B for 4 min. Initial conditions were returned over 2 min and then held at 100% solvent A to equilibrate for 3 min. The flow rate was 0.4 mL·min^−1^ for positive and 0.36 mL·min^−1^ for negative; injection volume was 2 μL and column oven temperature was 50°C. Polar metabolites were separated on HILIC Syncronis (100 × 2.1 mm, 1.9 μm particle size; Thermo Fisher Scientific (San Jose, CA, USA)) column with an in‐line filter. Metabolite separation in positive mode was performed using 0.1% formic acid in LC‐MS water (solvent A) and 0.1% formic acid in acetonitrile (solvent B). Negative mode used 10 mM ammonium acetate in LC‐MS water (solvent A) and LC‐MS acetonitrile (solvent B). The elution gradient for HILIC was as follows: isocratic at 95% solvent B for 1.5 min, 95%–40% solvent B in 10.5 min, maintained at 40% solvent B for 2 min, returned to initial conditions over 1 min, and then held for 5 min for column equilibration. The flow rate was 0.3 mL·min^−1^; injection volume 2 μL, and column oven temperature was 50°C.

Data was collected in full scan mode at a mass resolution of 70,000 (Full Width at Half Maximum, FWHM, at a m/z ratio of 200) over a scan range of m/z 70–1000. Tandem MS (MS/MS) was conducted on all QC samples using data‐dependent acquisition (DDA) in “discovery” mode with the following settings: resolution = 17,500, isolation width = 3.0 m/z, and stepped collision energy = 15 and 30 eV. Source and ion transfer conditions were set as follows: sheath gas = 35 (arbitrary units), auxiliary gas = 10 (arbitrary units), source heater = 350°C, capillary temperature = 350°C, ion spray voltage = 2.5 kV (negative ion mode) and 3.0 kV (positive ion mode). The automatic gain control target was set to 3 × 10^−6^. Xcalibur software (Thermo Fisher Scientific v4.3) was used for data acquisition. Prior to analysis, commercially purchased calibrant solutions (negative and positive ion calibration solutions) from Thermo Fisher Scientific were used to externally calibrate the Orbitrap mass spectrometer.

### 2.8 Data processing

Following data acquisition, raw data files from all modes were pre‐processed using Compound Discoverer™ 3.1 (Thermo Scientific, San Jose, CA, USA) within an untargeted metabolomics framework, encompassing HILIC POS, C18 POS, and C18 NEG analyses. Total ion chromatograms were aligned based on retention times (RT) using an adaptive curve, with a mass tolerance of 5 ppm and a maximum retention time shift of 0.5 min. Features with a signal‐to‐noise ratio >5 and an intensity ≥1,000,000 across each dataset were aggregated into compounds based on ion adducts. Ions identified in the blanks were subtracted from the sample data using the “mark background compounds” mode. Metabolite identification and annotation were performed in accordance with the Metabolomics Standards Initiative (MSI) guidelines (Sumner et al., 2007) and were annotated primarily using an in‐house library, including *mz*, MS/MS and retention time match. Analytical signal drift was corrected through quality control‐regularized spline correction (QC‐RSC) (Kirwan et al., 2013). In accordance with established protocols (Broadhurst et al., 2018), metabolites exhibiting an RSD_QC_ > 20% or a dispersion ratio (D‐ratio) > 30% were excluded from further statistical analysis due to inadequate precision.

### 2.9 Statistical analysis

Preprocessed metabolic data from LC‐MS positive and negative modes were combined for the purpose of statistical modeling. The statistical analysis of the dataset comprised a combination of multi‐ and univariate analyses. Multivariate analyses were employed to consider multiple outcome parameters (i.e., metabolites) and to allow for correlational patterns between parameters (Alonso et al., 2015). To investigate any intervention‐induced alterations in the metabolic response to the three dietary groups (i.e., HCHO, LEA, and LCHF) across all timepoints (i.e., Fasted, EXR‐pre, EXR‐post, EXR‐1h, and EXR‐3h), repeated measures ANOVA Simultaneous Component Analysis+ (RM‐ASCA+) was conducted using the ALASCA package in R. The RM‐ASCA+ method allows for a combination of general linear (mixed) models with Principal Component Analysis (PCA) and is particularly suitable for multivariate data from longitudinal studies (Cox et al., 2025; Govus et al., 2025; Jarmund et al., 2022). The RM‐ASCA+ models for analysis of the dietary conditions and exercise data were initialized as:

$$\text{Metabolite concentration}\sim \mathrm{TP}\times \mathrm{Grp}+\left(1|\mathrm{ID}\right)$$

The formula represents a linear mixed model with metabolite concentration as the outcome variable. “TP” refers to time point either in the harmonization or the intervention phase of the design. “Grp” denotes the group variable, “ID” is the participant identification number, which contributed to the model as a random effect. Within the ALASCA function, specification of scaling, separate effects, validation number, validation method, and dimension reduction were used. The scale function was set to “std1” to divide the values by the SD of all baseline samples for each metabolite. For instance, the values for Fasted, EXR‐pre, EXR‐post, EXR‐1h, and EXR‐3h were standardized with reference to baseline values attained from the harmonization phase. Bootstrapping was selected for the validation, which randomly selected participants with replacement. A scree plot was created to determine the number of principal components (PC) requiring further investigation. For each PC deemed relevant, the scores and loadings were visualized, and prediction plots were created for the 15 metabolites with the highest and lowest loadings on each PC of interest. To account for multiple testing, all univariate *p* values were adjusted using the Benjamini‐Hochberg False Discovery Rate (FDR) procedure, with significance defined by a *q*‐value <0.05. All figures related to RM‐ASCA+ modeling were created using the ALASCA package (Jarmund et al., 2022).

To further explore similarities in metabolite concentration patterns, Hierarchical Cluster Analysis (HCA) was performed on annotated metabolites using Ward's linkage method and Pearson's correlation coefficient as the similarity metric. Clusters were identified from the lowest linkage points in the resulting dendrogram, indicating metabolites with the most similar temporal responses. Identified metabolites within clusters were cross‐referenced against the Human Metabolome Database (HMDB; http://www.hmdb.ca) to determine their class, sub‐class, and known biological functions.

Substrate concentrations from capillary samples (glucose, lactate, and βHB) were assessed using two‐way ANOVA within dietary groups, while serum FFA concentrations were analyzed using a mixed‐effects model due to missing data points. Substrate data has previously been presented (Burke et al., 2021) without the addition of the LEA group. Tukey post hoc test was applied for pairwise comparisons where applicable. All data and statistical analyses were generated in RStudio v.4.3.3 (R Foundation for Statistical Computing, Viena, Austria, RRID:SCR_000432) and GraphPad Prism (Version 10.0.3, GraphPad Software Inc., La Jolla, CA, USA).

## 3 RESULTS

All participants complied with their assigned dietary interventions and in the monitoring of both food intake and training sessions. Participant characteristics are displayed in Table 2. There were no significant differences between groups for these characteristics.

**TABLE 2:** Subject characteristics at baseline testing.

Characteristic	HCHO (*n = 6*)	LEA (*n = 7*)	LCHF (*n = 7*)
Age (years)	31 ± 5	30 ± 2	27 ± 3
Body mass (kg)	66.2 ± 7.8	68.5 ± 6.4	66.2 ± 8.1
V̇O_2_peak (mL·kg^−1^·min^−1^)	63.9 ± 3.6	61.9 ± 5	67.7 ± 6.1

*Note*: Data presented as mean ± SD.

Abbreviations: HCHO, high energy‐high carbohydrate; LCHF, low carbohydrate‐high fat; LEA, low‐energy availability.

### 3.1 Exercise circulating blood metabolite responses

**FIGURE 2:**
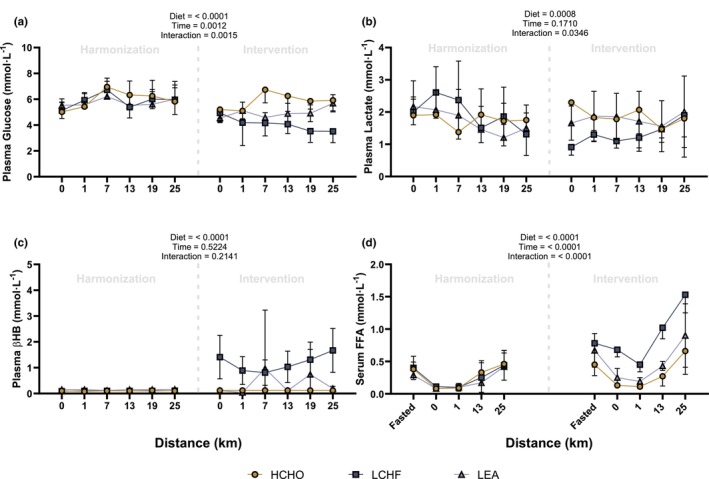
Line graphs of substrate analysis for capillary glucose (a), lactate (b), β‐Hydroxybutyrate [βHB] (c) and serum free‐fatty acid [FFA] (d). Harmonization represents the period in which all athletes consumed a high‐energy, high‐carbohydrate (HCHO) diet for 5 days. Following the harmonization period, athletes were allocated into either a HCHO, low‐energy availability (LEA) or low‐carbohydrate, high‐fat (LCHF) diet for 5 days (Intervention phase). Group, Time, and Interaction *p* values refer to main effects from the overall mixed‐effects model or two‐way ANOVA. Data presented as mean ± SD. Note that data from the LCHF and HCHO groups have previously been presented within other work from our research group (Burke et al., 2021).

There were no statistical differences detected between groups for metabolites assessed in capillary samples (glucose, lactate, and βHB) or serum (FFA) during the 25 km walk performed in the Harmonization phase (Figure 2a–d). However, following the separation into dietary treatment groups, there was a significant main effect of diet for blood glucose (Figure 2a), with concentrations higher in HCHO compared to both LEA and LCHF (*p* < 0.0001), and higher in LEA compared to LCHF. A significant main effect of diet was also observed for lactate (*p* = 0.0008, Figure 2b), where concentrations were lower in LCHF compared to both HCHO and LEA, with no difference between HCHO and LEA. For βHB, there was a significant main effect of diet (*p* < 0.0001, Figure 2c), with concentrations higher in LCHF compared to both HCHO and LEA, and no difference between HCHO and LEA. A significant main effect of diet was also observed for serum FFA concentrations (*p* < 0.0001, Figure 2d), with LCHF greater than both LEA and HCHO, and LEA greater than HCHO. Pairwise comparisons are shown in Table 3.

**TABLE 3:** Pairwise comparisons of substrates across dietary conditions during harmonization and intervention phases.

Timepoint								
Substrate	*p* Value comparison	Fasted	0 km	1 km	7 km	13 km	19 km	25 km
Harmonization								
Plasma Glucose	HCHO vs. LCHF	–	*p* = 1.00	*p* = 0.90	*p* = 1.00	*p* = 0.37	*p* = 1.00	*p* = 1.00
	HCHO vs. LEA	–	*p* = 0.90	*p* = 1.00	*p* = 0.64	*p* = 0.56	*p* = 0.76	*p* = 1.00
	LCHF vs. LEA	–	*p* = 0.95	*p* = 0.97	*p* = 0.87	*p* = 1.00	*p* = 0.95	*p* = 1.00
Plasma Lactate	HCHO vs. LCHF	–	*p* = 1.00	*p* = 0.45	*p* = 0.10	*p* = 0.89	*p* = 1.00	*p* = 0.85
	HCHO vs. LEA	–	*p* = 0.98	*p* = 1.00	*p* = 0.74	*p* = 0.83	*p* = 0.75	*p* = 0.99
	LCHF vs. LEA	–	*p* = 1.00	*p* = 0.67	*p* = 0.79	*p* = 1.00	*p* = 0.47	*p* = 1.00
Plasma βHB	HCHO vs. LCHF	–	*p* = 1.00	*p* = 1.00	*p* = 1.00	*p* = 1.00	*p* = 1.00	*p* = 1.00
	HCHO vs. LEA	–	*p* = 1.00	*p* = 1.00	*p* = 1.00	*p* = 1.00	*p* = 1.00	*p* = 1.00
	LCHF vs. LEA	–	*p* = 1.00	*p* = 1.00	*p* = 1.00	*p* = 1.00	*p* = 1.00	*p* = 1.00
Serum FFA	HCHO vs. LCHF	*p* = 1.00	*p* = 1.00	*p* = 1.00	–	*p* = 0.97	–	*p* = 1.00
	HCHO vs. LEA	*p* = 0.95	*p* = 1.00	*p* = 1.00	–	*p* = 0.64	–	*p* = 1.00
	LCHF vs. LEA	*p* = 0.88	*p* = 1.00	*p* = 1.00	–	*p* = 0.97	–	*p* = 1.00
Intervention								
Plasma Glucose	HCHO vs. LCHF	–	*p* = 0.99	*p* = 0.41	*p* < 0.001	*p* < 0.001	*p* < 0.001	*p* < 0.001
	HCHO vs. LEA	–	*p* = 0.74	*p* = 1.00	*p* = 0.001	*p* = 0.06	*p* = 0.37	*p* = 1.00
	LCHF vs. LEA	–	*p* = 0.95	*p* = 0.35	*p* = 0.94	*p* = 0.49	*p* = 0.04	*p* < 0.001
Plasma Lactate	HCHO vs. LCHF	–	*p* = 0.004	*p* = 0.72	*p* = 0.47	*p* = 0.21	*p* = 1.00	*p* = 1.00
	HCHO vs. LEA	–	*p* = 0.53	*p* = 1.00	*P* = 1.00	*p* = 0.93	*p* = 1.00	*p* = 0.99
	LCHF vs. LEA	–	*p* = 0.31	*p* = 0.62	*p* = 0.29	*p* = 0.74	*p* = 1.00	*p* = 1.00
Plasma β*HB*	HCHO vs. LCHF	–	*p* < 0.001	*p* = 0.07	*p* = 0.17	*p* = 0.02	*p* = 0.001	*p* < 0.001
	HCHO vs. LEA	–	*p* = 1.00	*p* = 1.00	*p* = 0.047	*p* = 1.00	*p* = 0.22	*p* = 1.00
	LCHF vs. LEA	–	*p* < 0.001	*p* = 0.03	*p* = 0.99	*p* = 0.012	*p* = 0.30	*p* < 0.001
Serum FFA	HCHO vs. LCHF	*p* = 0.03	*p* < 0.001	*p* = 0.02	–	*p* < 0.001	–	*p* < 0.001
	HCHO vs. LEA	*p* = 0.33	*p* = 0.86	*p* = 0.97	–	*p* = 0.64	–	*p* = 0.20
	LCHF vs. LEA	*p* = 0.91	*p* < 0.001	*p* = 0.10	–	*p* < 0.001	–	*p* < 0.001

*Note*: Data represent *p* values for pairwise comparisons at specific timepoints (Fasted to 25 km) during the Harmonization and Intervention phases. Statistical significance is defined as *p* < 0.05. *p* Values were determined using Tukey post hoc test.

Abbreviations: βHB, β‐hydroxybutyrate; FFA, free fatty acids; HCHO, high energy‐high carbohydrate; LCHF, low carbohydrate‐high fat; LEA, low‐energy availability.

### 3.2 RM‐ASCA+ analysis of plasma metabolomics data

**FIGURE 3:**
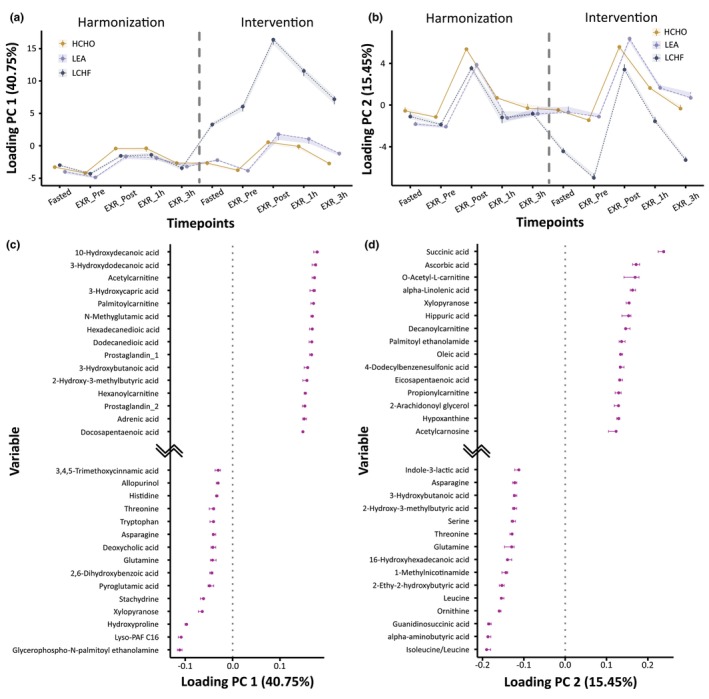
RM‐ASCA+ Model. Principal component analysis (PCA) score for principal component (PC) 1 (a) and PC 2 (b). Loading plots for PC 1 (c) and PC 2 (d) are also shown, identifying metabolites that drive group/time separation. Harmonization represents the period in which all athletes consumed a high‐energy, high‐carbohydrate (HCHO) diet for 5 days. Following the harmonization period, athletes were allocated into either a HCHO, low‐energy availability (LEA), or low‐carbohydrate, high‐fat (LCHF) diet for 5 days (Intervention phase).

A total of 5391 reproducible features from the preprocessing were extracted from the raw data, of which 138 were annotated and taken to the subsequent multivariate model. Using the annotated metabolites, the RM‐ASCA+ model was built using all time points containing the three groups. The model revealed metabolites that drive the differences observed between the dietary conditions across time within the domains of PC 1 and PC 2. Here, PC 1 explained 40.75% of the variance (Figure 3a) and PC 2 explained 15.45% of the variance (Figure 3b). The scores and loadings for each bootstrap iteration, as well as the confidence intervals of the loadings and the final data matrix, are shown in Figures S1 and S2 and Appendix S1. Metabolites with positive loadings on PC 1 of the RM‐ASCA+ model (Figure 3a) remained stable during the harmonization phase (i.e., timepoints “Fasted” through to “EXR‐3hr”) and then increased over time in the second phase of the study for the LCHF group, with small increases for both HCHO and LEA groups. While explaining less variance, PC 2 accounts for 15% of the variance between Fasted and EXR‐Post timepoints of the Intervention phase (Figure 3b). The separation between the LCHF group and HCHO and LEA is apparent at Fasted and EXR‐Pre timepoints (Figure 3b), while exercise from the 25 km racewalk ameliorates this separation.

Loading plots were generated to display the 15 metabolites with the largest positive and negative loadings (Figure 3c,d). Positive loadings on PC 1 were driven by long‐chain hydroxy‐acylcarnitines and FA, including 10‐hydroxydecanoic acid, 3‐hydroxydecanoic acid, acylcarnitine, and palmitoylcarnitine (Figure 3c). Metabolites that display the strongest contribution to the model observe the greatest shift towards the positive range along the x‐axis, with these metabolites increasing markedly following exercise in the LCHF condition, while remaining relatively unchanged in LEA and HCHO (Figure 3a). The metabolite exhibiting the strongest contribution for PC 1 was 10‐hydroxydecanoic acid (loading = 0.178, Figure 3c). Metabolites that display a negative shift along the x‐axis of the loading plot reflect a decreased loading score on the PC 1 score. Glycerophospho‐N‐palmitoyl ethanolamine (PC 1; Figure 3c) and isoleucine/leucine (PC 2; Figure 3d) displayed the strongest negative contribution, which was suppressed under LCHF relative to HCHO and LEA. Positive loadings on PC 2 were largely driven by succinic acid (loading = 0.238), ascorbic acid, O‐acetyl‐L‐carnitine, and α‐linolenic acid, which rose post exercise in the HCHO and LEA but were blunted under LCHF (Figure 3b,d).

### 3.3 Standardized 25 km endurance walk

**FIGURE 4:**
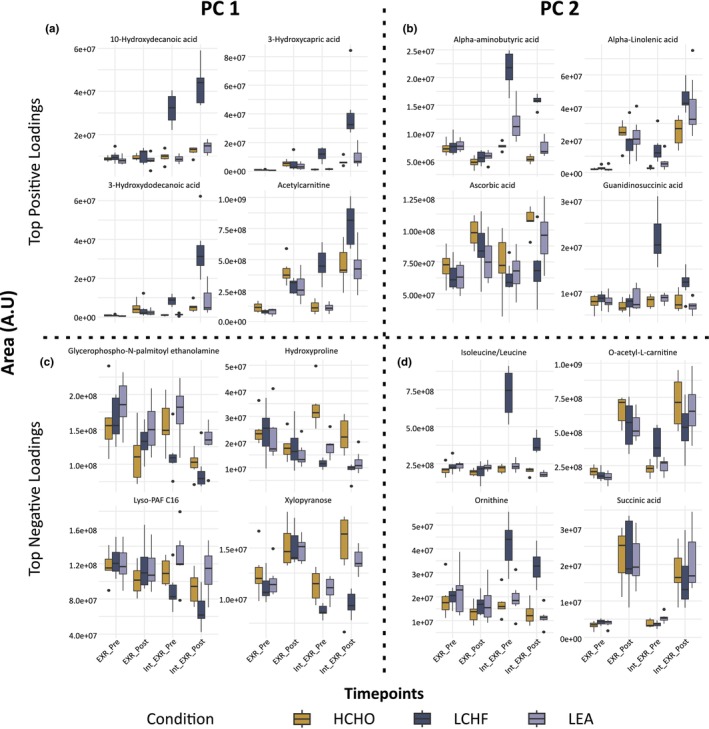
Box‐and‐whisker plots of the top four positive and negative loadings that contribute most to the separation of dietary conditions within the domains of PC 1 (a and c) and PC 2 (b and d). Here, selected metabolite response pre‐exercise (EXR‐Pre) and immediately post‐exercise (EXR‐Post) for the harmonization phase (EXR‐Pre and EXR‐Post) and the Intervention phase (Int_EXR‐Pre and Int_EXR‐Post) are displayed. Box‐and‐whisker plots represent the median (bold horizontal line), the interquartile range (box), and the minimum and maximum values excluding outliers (whiskers). Individual data points plotted beyond the whiskers represent outliers.

The greatest separation of dietary groups occurred in response to the 25 km racewalk (between EXR‐pre and EXR‐post). To illustrate this, the top four positive and negative loadings from PC 1 and PC 2 are shown as box‐and‐whisker plots (Figure 4). For PC 1, the strongest positive loadings were 10‐hydroxydecanoic acid, 3‐hydroxydodecanoic acid, acetylcarnitine, and 3‐hydroxycapric acid (Figure 4a), while glycerophospho‐N‐palmitoyl ethanolamine, lyso‐PAF C16, hydroxyproline, and xylopyranose were the strongest negative loadings (Figure 4c). For PC 2, succinic acid, ascorbic acid, O‐acetyl‐L‐carnitine, and alpha‐linolenic acid contributed most positively (Figure 4b), whereas isoleucine/leucine, alpha‐aminobutyric acid, guanidinosuccinic acid, and ornithine contributed most negatively (Figure 4d). Furthermore, to assist in the interpretation of these findings, the biological roles, functions, and implications for fuel viability of the key plasma metabolites identified in Figure 4 are summarized in Table 4.

**TABLE 4:** Biological roles, functions, and observed responses of prominent plasma metabolites.

Metabolite class	Representative metabolites	Biological role and function	Observed response	Metabolic significance and fuel viability
Medium‐Chain Hydroxy Fatty Acids	10‐Hydroxydecanoic acid 3‐Hydroxycapric acid 3‐Hydroxydodecanoic acid	Intermediates produced during fatty acid β‐oxidation; indicators of lipid turnover.	Higher abundance observed in LCHF post‐exercise; remained relatively stable in HCHO and LEA.	Potentially indicates a shift towards lipid‐based metabolism as a primary fuel source during CHO scarcity.
Acylcarnitines	Acetylcarnitine O‐acetyl‐L‐carnitine	Facilitate fatty acid transport; O‐acetyl‐L‐carnitine buffers excess acetyl‐CoA to maintain mitochondrial CoASH pools.	Acetylcarnitine levels appeared higher in LCHF; O‐acetyl‐L‐carnitine levels showed post‐exercise elevation in HCHO/LEA but appeared lower in LCHF.	May reflect the balance between increased glycolytic flux (HCHO/LEA) and an increased reliance on lipid oxidation (LCHF).
Amino Acids and Derivatives	Isoleucine/Leucine Alpha‐aminobutyric acid Ornithine Hydroxyproline	Essential branched‐chain amino acids (BCAAs) and urea cycle intermediates involved in protein metabolism.	Showed divergent patterns in LCHF; these metabolites contributed to model separation between dietary groups.	May indicate variations in amino acid catabolism or shifts in nitrogen handling/gluconeogenesis during CHO restriction.
TCA Cycle Intermediates	Succinic acid	A critical catalytic intermediate in the Citric Acid Cycle for aerobic ATP production.	Elevated levels observed in HCHO and LEA post‐exercise; levels appeared lower in the LCHF condition.	Suggests that LCHF conditions may influence aerobic energy flux or anaplerotic support compared to CHO‐supported states.
Lipid Signaling and Phospholipids	Glycerophospho‐N‐palmitoyl ethanolamine Lyso‐PAF C16	Structural components of membranes and signaling molecules involved in cellular stress/remodeling.	Observed lower levels in LCHF compared to HCHO and LEA groups.	May reflect systemic alterations in lipid membrane responses or signaling pathways under restricted CHO conditions.
Other Factors	Ascorbic acid Alpha‐Linolenic acid Xylopyranose Guanidinosuccinic acid	Antioxidants, essential fatty acids, and various metabolic by‐products.	Ascorbic acid and Alpha‐Linolenic acid showed higher post‐exercise levels in HCHO/LEA than in LCHF.	May indicate that dietary availability potentially influences acute antioxidant responses and essential fatty acid availability during exercise.

Abbreviations: CHO, carbohydrates; CoASH, coenzyme A; EA, energy availability; HCHO, high‐energy, high‐carbohydrate; LCHF, low‐carbohydrate, high‐fat diet; LEA, low energy availability diet; TCA, tricarboxylic acid cycle.

### 3.4 Hierarchical clustering analysis

**FIGURE 5:**
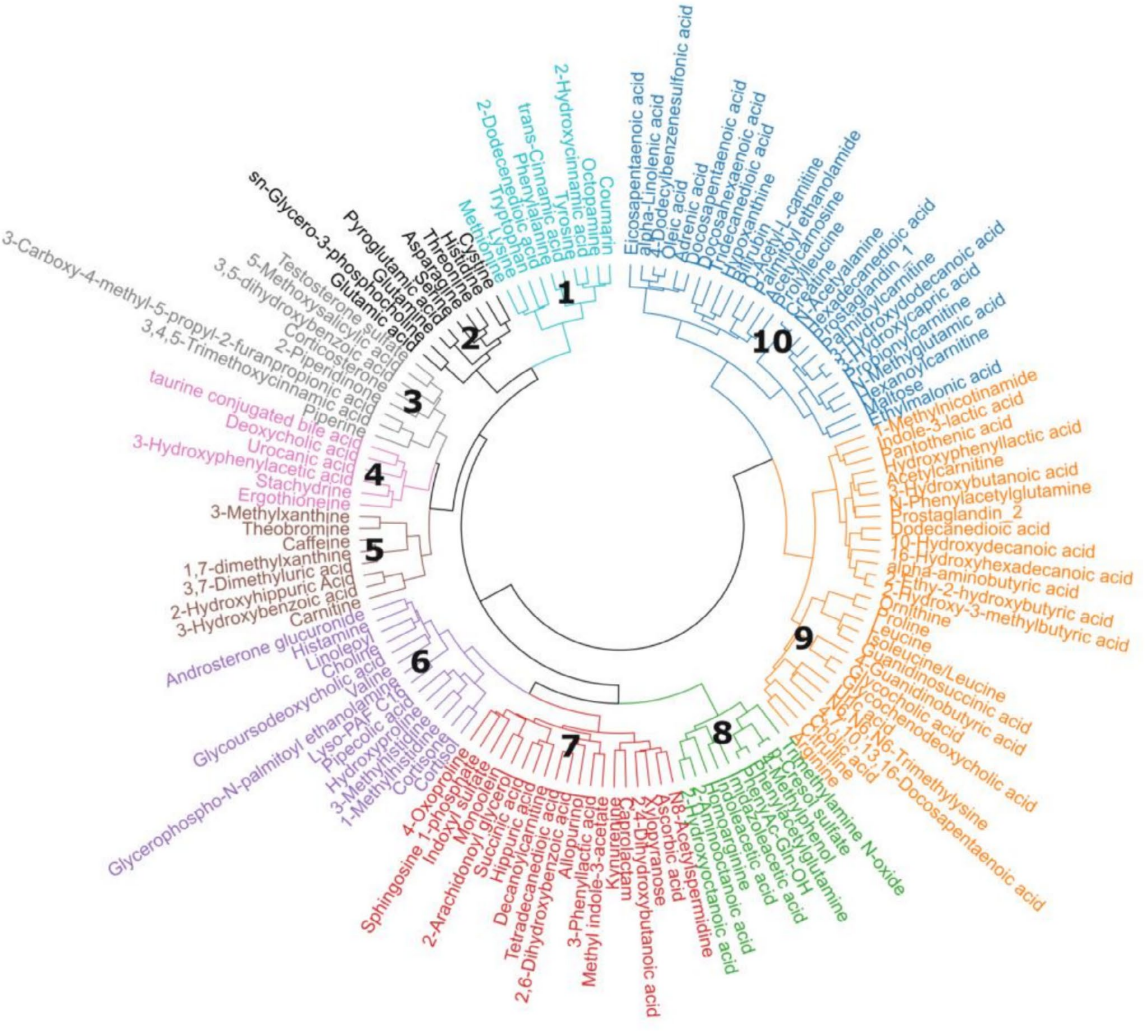
Hierarchical cluster analysis (HCA) dendrogram of annotated metabolites from plasma. Metabolites were clustered based on similar abundance patterns and categorized according to subclass species. Ten clusters (labeled 1–10) were identified. The lowest linkages within the HCA dendrogram indicate metabolites that display similar relative responses between the experimental groups.

Using hierarchical clustering on the annotated metabolites, 10 distinct clusters were formed and are displayed as a dendrogram (Figure 5). Using the HMDB, annotated metabolites were cross‐referenced so that metabolite class, subclass, and function could be extracted and that clusters could be assigned to a metabolite grouping. The following clusters were identified: Cluster 1 (aromatic and amino acids); Cluster 2 (amino acids and derivatives); Cluster 3 (steroid hormones, phenolic acids, and alkaloid‐related compounds); Cluster 4 (bile acid metabolism, amino acid metabolism, and aromatic acid derivatives); Cluster 5 (purines and derivatives); Cluster 6 (amino acid derivatives, steroid hormones, and lipid conjugates); Cluster 7 (lipids, amino acids, and microbial metabolites); Cluster 8 (microbial and amino acid‐derived metabolites); Cluster 9 (amino acids, bile acids, fatty acids (FA), and microbial catabolites); and Cluster 10 (fatty acyls, hydroxy acids, and lipid signaling metabolites). By grouping the clusters, it was possible to determine similar trajectories most impacted by the dietary interventions. Nine of the top 15 positive loadings from PC 1 were found in Cluster 10 (fatty acyls, hydroxy acids, and lipid signaling metabolites), whereas 11 of the top 15 negative loadings from PC 2 were from Cluster 9 (amino acids, bile acids, and microbial catabolites).

## 4 DISCUSSION

We evaluated the impact of a short‐term (5 days) dietary intervention restricting either CHO (LCHF) or energy (LEA) in elite male race walkers undertaking a prolonged exercise protocol using an untargeted metabolic profiling approach. We detected >5000 metabolites via MS‐based metabolomics analysis and identified 138 metabolites in response to the LCHF and LEA conditions. Notably, clusters enriched in fatty acyls and hydroxy acid derivatives were significantly increased in athletes consuming the LCHF diet, with marked differences in metabolite profiles most evident during the recovery from a prolonged, strenuous, standardized exercise bout. In contrast, despite being energy‐deficient, the LEA group exhibited similar metabolic profiles to those of HCHO. While the data presented here further contributes to a growing body of literature that describes the effects of a LCHF diet, these findings support our and other previous observations that CHO restriction may underpin changes to metabolic biomarkers rather than energy restriction per se (Fensham et al., 2022; McKay, Peeling, et al., 2022).

This study provided a real‐world investigation of the effects of severe CHO restriction (LCHF vs. HCHO) on metabolic characteristics, as well as a comparison to LEA in which a substantial reduction in EI was also accompanied by a partial reduction in CHO. The study design did not allow a clean comparison of the isolated effects of EA or macronutrient intake. Indeed, it would be difficult to attribute the metabolic effects of the LCHF group solely to CHO restriction rather than high fat intake. Such a conclusion would require multiple iterations of diets that are artificial rather than “real world”. Indeed, we recognize that the effects of the LCHF diet could be driven by the very high fat intake in addition to the restriction of CHO availability. The LEA diet was constructed to standardize protein intake, then maximize CHO intake within the energy targets and everyday food choices. In LEA research (Jurov et al., 2022; Martin et al., 2021; McKay, Peeling, et al., 2022), reductions in EA are accompanied by variable decreases in CHO availability, which should be considered a potential moderator of the effects of LEA itself. This is important in gaining insight into scenarios of LEA that are at high risk of causing perturbations to some or all body systems (Burke, Ackerman, et al., 2023). The results of the current study indicate that changes in CHO availability are a key driver of the observed metabolic responses to diet and exercise rather than reductions in EA.

Multivariate analysis (ALASCA) revealed that the combination of PC 1 and PC 2 explained ~60% of the variance in circulating metabolites between dietary conditions, with the greatest divergence in the post‐exercise period. The LCHF condition was characterized by significant changes in multiple fatty acyl species and hydroxy acids derivatives immediately post exercise (EXR‐post), alongside increases in βHB and serum FFA. Such perturbations were not observed in either the LEA or HCHO groups (Figure 2c). These findings are in agreement with previous work by Helge et al. (2001) who demonstrated a greater rate of appearance of FA in arterial blood during moderate‐intensity exercise (~68% V̇O₂max) following 7 weeks of adaptation to a LCHF diet (62% fat and 21% CHO) compared to an isoenergetic CHO‐rich diet (20% fat and 65% CHO). The elevation of plasma FA conjugates and serum FFA detected in the LCHF group supports the concept of increased lipid mobilization during exercise following a CHO restricted diet (Helge et al., 2001). We have previously reported that adherence to a 5‐day LCHF diet increased rates of whole‐body fat oxidation during a four‐stage submaximal test in elite athletes (Burke et al., 2021), while others have observed a greater reliance on fat metabolism as well as concomitant increases in plasma FFA and βHB concentrations following a 3‐day LCHF diet (5% CHO, 73% fat, and 22% protein) (Peters et al., 2001). Crucially, however, this enhanced capacity for fat utilization does not translate to a performance advantage, with previously published results from this cohort demonstrating that 25 km racewalk times were significantly impaired (~3.4% slower) in the LCHF group compared to HCHO (Burke et al., 2021). It should be noted that caffeine was detected (cluster 5) and was likely attributed to intra‐exercise gels (containing 50 mg). While caffeine can stimulate lipolysis, the low, optional dose used here is unlikely to have meaningfully influenced the major lipid‐related metabolite patterns observed.

In contrast, the LEA condition did not elicit comparable alterations in lipid profiles, despite a drastic reduction in EI (~60%), accompanied by a partial decrease in CHO intake. One potential explanation is that CHO availability in LEA may not have reached the level of depletion required to provoke the marked shifts in lipid‐related metabolites as seen in LCHF. The “glycogen threshold” hypothesis proposes that intramuscular glycogen must decline below a defined level before initiating adaptive signaling cascades that favor enhanced lipid mobilization and ketogenesis (Impey et al., 2018). Although muscle glycogen was not directly assessed in the present study, the restriction of CHO in the LCHF group compared to the LEA group makes it possible that this threshold was surpassed, thereby driving the distinct accumulation of fatty acyls and hydroxy acid derivatives observed. In this context, glycogen functions not solely as a metabolic substrate but also as a regulatory signal that influences substrate partitioning (Lane et al., 2015). From an evolutionary perspective, such nutrient‐sensing mechanisms may have conferred a survival advantage by facilitating a rapid switch to lipid‐based fuels under conditions of CHO scarcity, independent of total EA. As such, differential glycogen availability between interventions could help explain, in part, the divergent metabolomic profiles observed. However, because the LCHF diet simultaneously manipulates both low CHO availability and high fat intake, the present data cannot distinguish between effects driven by CHO restriction per se versus elevated fat provision. As such, the findings should be interpreted as reflecting the combined influence of both factors rather than the isolated impact of CHO availability. During the post‐exercise recovery period, the LCHF group displayed increases in dicarboxylic acids (e.g., hexadecanedioic acid), medium‐chain FA (e.g., 3‐hydroxydodecanoic acid and 3‐hydroxycapric acid), and long‐chain FA (e.g., adrenic acid). Such observations are consistent with others who have reported an upregulation of these metabolites which are indicative of increased reliance on β‐oxidation (Lehmann et al., 2010). Substrate selection is a tightly regulated process, largely controlled by the multienzyme complex pyruvate dehydrogenase (PDH), which is the rate‐limiting step for entry of CHO‐derived substrate into the mitochondria and the subsequent production of reducing equivalents by the TCA cycle. Short‐term adherence to a 3‐day LCHF diet (5% CHO and 73% fat) results in rapid increases in the activity and expression of PDH kinase, which phosphorylates and inhibits the PDH complex, thereby decreasing overall flux (Peters et al., 2001). In the context of athletic performance, PDH activity remains suppressed following a LCHF diet during exercise even after 24 h of restored CHO intake (Stellingwerff et al., 2006), indicating a sustained reduction in the capacity to oxidize CHO and a potential performance limitation. Chronic LCHF adherence also leads to reduced glucose tolerance, insulin receptor substrate 1, and GLUT4 protein content in well‐trained individuals (Webster et al., 2020), further highlighting that CHO availability is essential for maintaining the capacity for energy production via glycolytic pathways. A reduced PDH flux may also diminish anaplerotic support for the TCA cycle and contribute to declines in intermediate pool size. Although TCA cycle intermediates can fall by more than 50% during prolonged (~90 min) exercise, oxidative capacity is usually preserved through compensatory pathways, including amino acid catabolism and FA‐derived anaplerosis (Gibala et al., 2002). Although PDH activity and glycolytic flux were not directly measured, the distinct accumulation of dicarboxylic acids, hydroxy FA derivatives, and acylcarnitines in the LCHF group is consistent with suppressed PDH flux limiting CHO oxidation and reinforcing reliance on β‐oxidation during and after the 25 km walk. While our data cannot establish causality, the metabolite profiles align with prior work showing that PDH inhibition under LCHF conditions promotes lipid‐based fuel selection. In addition to this regulatory role of PDH, the elevations in lipid intermediates also suggest that downstream processes, including FA transport and β‐oxidation, may be impacted by CHO restriction (Howard & Margolis, 2020).

The 25 km endurance walk was a major driver of group separation in the ALASCA scores, with effects most pronounced during recovery. As expected, exercise‐induced shifts in substrate metabolism across all conditions, including elevations in 3‐hydroxybutanoic acid, FFA, and several long‐chain FA such as palmitoylcarnitine, docosapentaenoic acid, and adrenic acid. These changes were most pronounced in the LCHF group, where the persistent elevation throughout recovery suggests sustained rates of lipolysis to meet muscle demands. Consistent with our findings, a previous study employing untargeted metabolomics reported a nine‐fold increase in medium‐ and long‐chain acylcarnitines (8‐, 10‐, and 12‐carbon length species) immediately after prolonged, moderate‐intensity exercise (~75% V̇O_2_max) (Lehmann et al., 2010). Here, the presence of O‐acetyl‐L‐carnitine further indicates a shift towards fat‐based fuels for oxidation and reduced glycolytic flux (Lehmann et al., 2010), consistent with the lower lactate concentrations. In this regard, it has been suggested that CHO, rather than fat availability, determines the rate of lipid oxidation (Schrauwen et al., 2000; Sidossis et al., 1996). Nevertheless, while accumulation of FA likely depicts an increase in lipid oxidation, accumulation of O‐acetyl‐L‐carnitine is more likely to reflect excess acetyl‐CoA and functions as a buffering system to maintain the mitochondrial CoASH pool. By transferring acyl groups onto carnitine, mitochondria preserve CoASH availability to sustain ongoing oxidative metabolism. Indeed, when acylcarnitines accumulate beyond mitochondrial handling capacity, they can spill over into circulation (Lehmann et al., 2010), which explains their detection in plasma. This highlights the complexity of interpreting plasma metabolomics data, as elevations may represent both increased lipid oxidation and incomplete substrate utilization.

Restriction of CHO may also impair mitochondrial function by promoting the accumulation of lipid intermediates. For example, increased intramuscular triglycerides (IMTG) and palmitoyl‐CoA have been shown to inhibit ADP transport, thereby constraining ATP production and contributing to incomplete FA oxidation (Bachmann et al., 2001; Ho & Pande, 1974; Leckey et al., 2018; Ludzki et al., 2015; Zderic et al., 2004). In parallel, reduced availability of CHO‐derived intermediates such as oxaloacetate can limit TCA cycle flux, restricting acetyl‐CoA entry and promoting feedback inhibition. Together, these mechanisms may overwhelm mitochondrial oxidative capacity, resulting in spillover of intermediates, including hydroxylated FA and acylcarnitines, into the circulation (Koves et al., 2008). However, such a hypothesis remains speculative and would require further investigations to uncover these mechanisms.

Despite serving an exploratory and “hypothesis‐generating” role, a major limitation of metabolomics is that it cannot determine the origin of detected metabolites. Indeed, such information is essential for drawing conclusions about metabolic pathways that may be implicated due to divergent dietary conditions (i.e., LCHF vs. LEA). In this regard, other pathways and systems may have been affected by the dietary conditions. While only explaining ~15% of the variance in the separation between dietary conditions, PC 2 displayed reduced loading scores for HCHO and LEA which were driven primarily by metabolites implicated in amino acid catabolism, nitrogen handling, and the urea cycle. Indeed, untargeted metabolomics measures steady‐state metabolite abundances rather than metabolic flux. Consequently, elevations or reductions in circulating metabolites cannot be interpreted as evidence of increased pathway activity, altered substrate oxidation, or enhanced mitochondrial function. Without isotopic tracer methods or direct measures of enzymatic flux, we are unable to determine whether observed differences reflect changes in production, utilization, transport, or tissue release. Moreover, the divergence of EXR‐post metabolite profiles may partially reflect the last meal effect, rather than solely chronic adaptations to the dietary conditions. Furthermore, we acknowledge that this study utilized a small elite male athlete cohort. Because female athletes often exhibit distinct substrate utilization patterns during exercise and may demonstrate different metabolic sensitivity to energy deficits, these findings may not be generalizable across sexes. Thus, the observed changes likely represent the combined influence of both high fat intake and low CHO availability within this specific group. Further investigations incorporating larger, sex‐diverse cohorts are required to clarify how these alterations relate to broader perturbations in metabolic health as well as whether chronic LEA adaptation exposes greater metabolic perturbations compared to those observed here. Lastly, metabolite identification in untargeted metabolomics is constrained by the limited spectral libraries. Many detected features cannot be matched to known metabolites with a high degree of confidence, and structural isomers may remain indistinguishable without further targeted validation.

In conclusion, the findings from the current study indicate that CHO restriction rather than LEA may induce greater metabolic perturbations in elite athletes undergoing strenuous, endurance‐based exercise. These results provide a foundation for subsequent investigations and underscore the importance of further characterizing lipid metabolic responses to CHO restriction and LEA using a combination of targeted metabolomic and lipidomic techniques. Understanding these metabolic responses may be crucial for athletes and sports practitioners alike, as dietary strategies involving CHO restriction may inadvertently impair recovery or long‐term health when implemented during periods of sustained LEA.

## AUTHOR CONTRIBUTIONS

This study was conducted at the Australian Institute of Sport, Canberra, Australia. Conception and design of the experiments was undertaken by J.W., A.K.A.M., and L.M.B. Collection, assembly, analysis, and interpretation of data was undertaken by K.A.D., N.G.L., J.W., A.K.A.M., M.L.R., D.B., S.N.R., N.T., L.M.B., and J.A.H. Drafting the article or revising it critically for important intellectual content was undertaken by K.A.D., N.G.L., J.W., L.M.B., and J.A.H. All authors have read and approved the final version of this manuscript and agree to be accountable for all aspects of the work in ensuring that questions related to the accuracy or integrity of any part of the work are appropriately investigated and resolved. All persons designated as authors qualify for authorship, and all those who qualify for authorship are listed.

## FUNDING INFORMATION

This study was funded by a grant from Australian Catholic University Research Fund (ACURF, 2017000034) to Louise Burke and John Hawley.

## CONFLICT OF INTEREST STATEMENT

None of the authors of this paper have a competing interest.

## DISCLAIMERS

The content is solely the responsibility of the authors and does not necessarily represent the official views of the Australian Catholic University.

## Supporting information


Figure S1.

Figure S2.

Appendix S1.

## Data Availability

The data that support the findings of this study are available from the corresponding author upon reasonable request.
