# Supplementary material for: Carbohydrate restriction drives greater perturbations in circulating metabolites than low energy availability in elite male athletes
Source: Physiol Rep. 2026 Feb 3;14(3):e70752. doi: 10.14814/phy2.70752 (PMC12868390; doi:10.14814/phy2.70752)
Supplement: Supplementary file 1 — Figure S1. [file PHY2-14-e70752-s001.docx]

Supplementary File S1 - Bootstrap iterations PC1 Panel A: Stability of Principal Component 1 Loadings across Conditions. This profile plot illustrates the stability of the loading scores for PC1, which explains 40.45% of the total variance. Solid lines represent the mean loading across experimental timepoints for the CON (orange), LEA (purple), and LCHF (green) conditions. The faint, dashed background lines represent individual bootstrap iterations, providing a visual measure of the component's robustness across resampled datasets. Panel B: Metabolite Loading Distributions and 95% Confidence Intervals. This forest plot ranks metabolites by their contribution to PC1. Grey points represent the distribution of individual bootstrap iterations, while the solid black horizontal bars indicate the 95% confidence intervals.
