# Supplementary material for: Carbohydrate restriction drives greater perturbations in circulating metabolites than low energy availability in elite male athletes
Source: Physiol Rep. 2026 Feb 3;14(3):e70752. doi: 10.14814/phy2.70752 (PMC12868390; doi:10.14814/phy2.70752)
Supplement: Supplementary file 3 — Appendix S1. [file PHY2-14-e70752-s003.zip › Supplementary File S3.docx]

Supplementary File S3 - Final Data Matrix Final Data Matrix contains the curated and normalized metabolomic concentrations used for all statistical analyses and visualizations in this study.
